# Fully-automated atrophy segmentation in dry age-related macular degeneration in optical coherence tomography

**DOI:** 10.1038/s41598-021-01227-0

**Published:** 2021-11-08

**Authors:** Yasmine Derradji, Agata Mosinska, Stefanos Apostolopoulos, Carlos Ciller, Sandro De Zanet, Irmela Mantel

**Affiliations:** 1 Department of Ophthalmology, University of Lausanne, Jules Gonin Eye Hospital, Foundation Asile des Aveugles, 15 Avenue de France, CP 5143, CH-1004 Lausanne, Switzerland; 2 RetinAI Medical AG, Freiburgstrasse 3, CH-3010 Bern, Switzerland

**Keywords:** Biomedical engineering, Macular degeneration

## Abstract

Age-related macular degeneration (AMD) is a progressive retinal disease, causing vision loss. A more detailed characterization of its atrophic form became possible thanks to the introduction of Optical Coherence Tomography (OCT). However, manual atrophy quantification in 3D retinal scans is a tedious task and prevents taking full advantage of the accurate retina depiction. In this study we developed a fully automated algorithm segmenting Retinal Pigment Epithelial and Outer Retinal Atrophy (RORA) in dry AMD on macular OCT. 62 SD-OCT scans from eyes with atrophic AMD (57 patients) were collected and split into train and test sets. The training set was used to develop a Convolutional Neural Network (CNN). The performance of the algorithm was established by cross validation and comparison to the test set with ground-truth annotated by two graders. Additionally, the effect of using retinal layer segmentation during training was investigated. The algorithm achieved mean Dice scores of 0.881 and 0.844, sensitivity of 0.850 and 0.915 and precision of 0.928 and 0.799 in comparison with Expert 1 and Expert 2, respectively. Using retinal layer segmentation improved the model performance. The proposed model identified RORA with performance matching human experts. It has a potential to rapidly identify atrophy with high consistency.

## Introduction

Age-related macular degeneration (AMD) is a chronic and progressive disease, with central visual loss in more advanced stages. Atrophic AMD is one of the late stage forms of the disorder, characterized by irreversible loss of retinal layers—photoreceptors, the retinal pigment epithelium (RPE), and the choriocapillaris^1^. Its traditional assessment is based on fundus autofluorescence (FAF) and color fundus photography (CFP).

**Figure 1:**
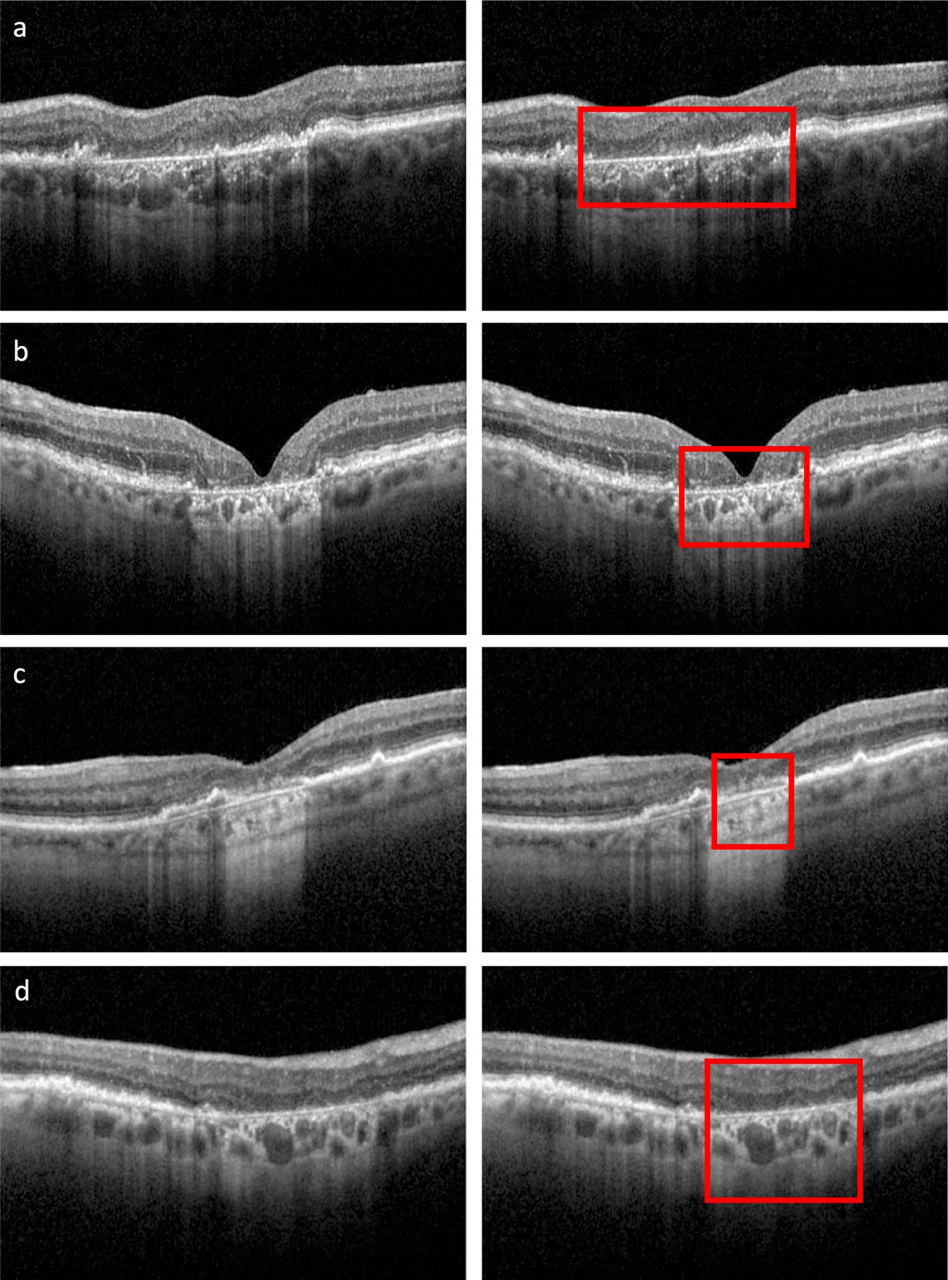
Examples of an OCT b-scan (left) and corresponding manual RORA segmentation (right). (**a**) Hypertransmission in the choroid is present within the annotated region. The status of the RPE is attenuated in the right part of the square and absent in the left part. (**b**) Clear presence of hypertransmission in the choroid, absence of the RPE line, and severe alteration of the photoreceptors. (**c**) Clearly altered but still present RPE layer within the annotated region. The RORA criteria are not fulfilled for the region to the left of the square, as there is incomplete hypertransmission and elevated RPE without significant attenuation. (**d**) Example of weak hypertransmission despite absence of the retinal pigment epithelium and existing RORA.

Recently, a new classification system for atrophy in AMD has been proposed, based on spectral-domain optical coherence tomography (SD-OCT)^2,3^—a modality providing 3D depiction of the retina at micrometer resolution. It allows for differentiating the affected retinal layers (*outer retinal atrophy* (ORA) versus *retinal pigment epithelium and outer retinal atrophy* (RORA)) as well as for distinguishing the completeness of the atrophic changes and as a result a more detailed pathology characteristics. Indeed, atrophic presentation in AMD is variable in size, progression, sharpness of borders, and the involved retinal layers as shown in Fig. 1. An OCT-based classification system allows accounting for those structural details and is no longer restricted to only the typically well-defined geographic atrophy in atrophic AMD seen in FAF images.

Deep learning (DL) applied in the medical domain enabled automated detection of different structures by extracting patterns from large quantities of annotated data. Among the various DL methods, Convolutional Neural Networks (CNNs) have been shown to be particularly well-suited to the field of medical imaging^4–7^. AI has been successfully applied also to the automated identification of geographic atrophy (GA) based on SD-OCT^8–16^. While the results of the previous studies were promising, they were not based on the most recent atrophy definition of RORA and the model's prediction relied primarily on the hypertransmission (increased signal) in the choroid, ignoring subtle changes in the photoreceptors and the RPE layer, which are critical for early atrophy detection.

The aim of our study was to develop a fully-automated algorithm to detect and measure RORA in macular SD-OCT volume scans according to the most recent definition^2,3^. To this end, a DL model based on CNN was developed. In contrast to other methods, it relies not only on choroid hypertransmission, which is insufficient to detect RORA as shown in Fig. 1D, but uses the information from full retina cross-section, additionally focusing on RORA-relevant retinal layers.

The interobserver agreement for RORA based on OCT *only* is challenging^2^, and an automated algorithm could allow for highly reproducible and rapid image analysis, which is potentially useful not only for research, but also for clinical practice.

## Results

The 62 OCT volumes were extracted from 62 eyes (43 right eyes, 19 left eyes) with atrophic age-related macular degeneration of 57 patients (mean age 85 ± 12 years, 38 females (66.6%)). The training set, used for development and cross validation, contained 2557 b-scans (44 cube scans), including 1166 b-scans without RORA and 1391 b-scans with RORA presence. The separate test set of 18 OCT volumes (each graded by two clinicians), contained 1038 b-scans, including 344 b-scans without RORA and 694 b-scans with RORA presence. An additional set of 5 healthy subjects (173 b-scans) was used to assess the false positive rate.

### Segmentation performance metrics

**Figure 2:**
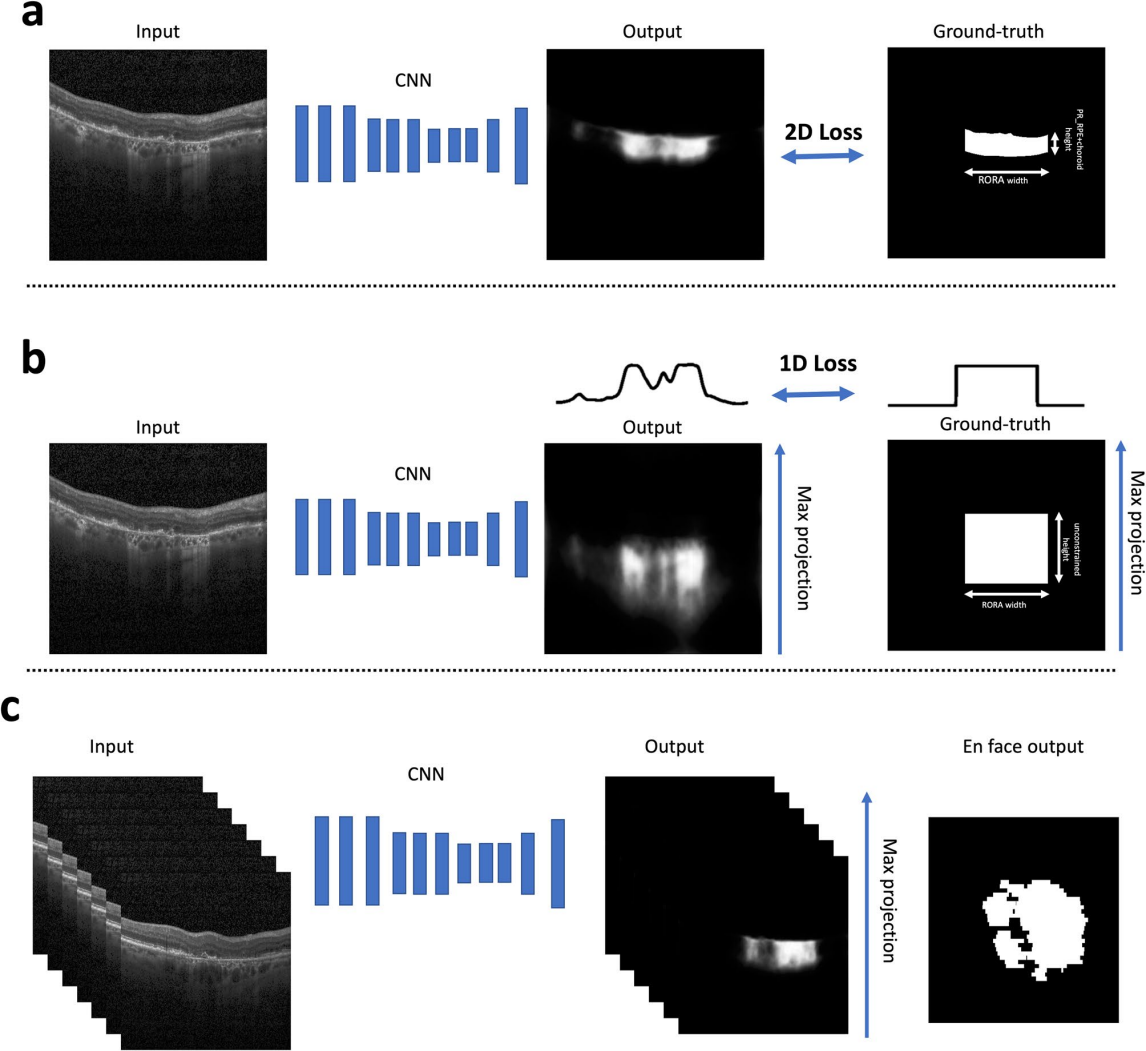
Method design. (**a**) A schematic illustration of our training method *with* layer segmentation prior. An input to the neural network is an OCT b-scan. The CNN output is a probability map for an atrophic region with a vertical span corresponding to the RPE layer and choroid. The loss is computed in 2d between the prediction and the RORA ground-truth masked with RPE and choroid. (**b**) A schematic illustration of the training approach *without* using layer segmentation prior. As the vertical span of the ground-truth bounding box is undefined, the loss is computed in 1d between the maximum probability projections of prediction and ground-truth. (**c**) Inference workflow—each b-scan in a test volume is fed to the CNN, which outputs RORA prediction. In order to obtain an en *face* view, the predictions are max-projected and thresholded at 0.5.

The performance metrics of the algorithm described in detail in “Methods” section and shown in Fig. 2, are summarized in Table 1 (cross validation) and Table 2 (separate test set graded by two experts). The segmentation metrics were computed per OCT scan and averaged.

**Table 1:** Mean performance metrics of our method on fivefold cross validation.

	Dice score	Precision	Recall	Kappa
Fold 1	0.886 ± 0.077	0.849 ± 0.122	0.941 ± 0.064	0.861 ± 0.080
Fold 2	0.853 ± 0.111	0.814 ± 0.171	0.924 ± 0.051	0.819 ± 0.158
Fold 3	0.928 ± 0.051	0.934 ± 0.056	0.926 ± 0.077	0.902 ± 0.063
Fold 4	0.888 ± 0.048	0.862 ± 0.074	0.920 ± 0.062	0.862 ± 0.065
Fold 5	0.859 ± 0.184	0.809 ± 0.214	0.954 ± 0.038	0.836 ± 0.185

**Table 2:** Mean performance metrics of the convolutional neural network (CNN) and graders on the separate test set.

	Dice score	Precision	Recall	Kappa
**Using annotations of grader 1 as ground-truth**				
Ji et al.^12^	0.716 ± 0.191	0.772 ± 0.244	0.727 ± 0.187	0.649 ± 0.180
Ours w/o layer prior	0.841 ± 0.114	**0.955 ± 0.050**	0.765 ± 0.157	0.801 ± 0.110
Ours with layer prior	**0.881 ± 0.074**	0.928 ± 0.054	**0.850 ± 0.119**	**0.846 ± 0.072**
Grader 2	0.863 ± 0.099	0.982 ± 0.014	0.782 ± 0.145	0.828 ± 0.100
**Using annotations of Grader 2 as ground-truth**				
Ji et al.^12^	0.677 ± 0.206	0.679 ± 0.264	0.770 ± 0.177	0.608 ± 0.188
Ours w/o layer prior	0.831 ± 0.090	**0.845 ± 0.134**	0.845 ± 0.130	**0.800 ± 0.086**
Ours with layer prior	**0.844 ± 0.076**	0.799 ± 0.133	**0.915 ± 0.064**	**0.800 ± 0.082**
Grader 1	0.863 ± 0.099	0.782 ± 0.145	0.982 ± 0.137	0.828 ± 0.100

Bold indicates the best performance values per evaluation parameter.

**Figure 3:**
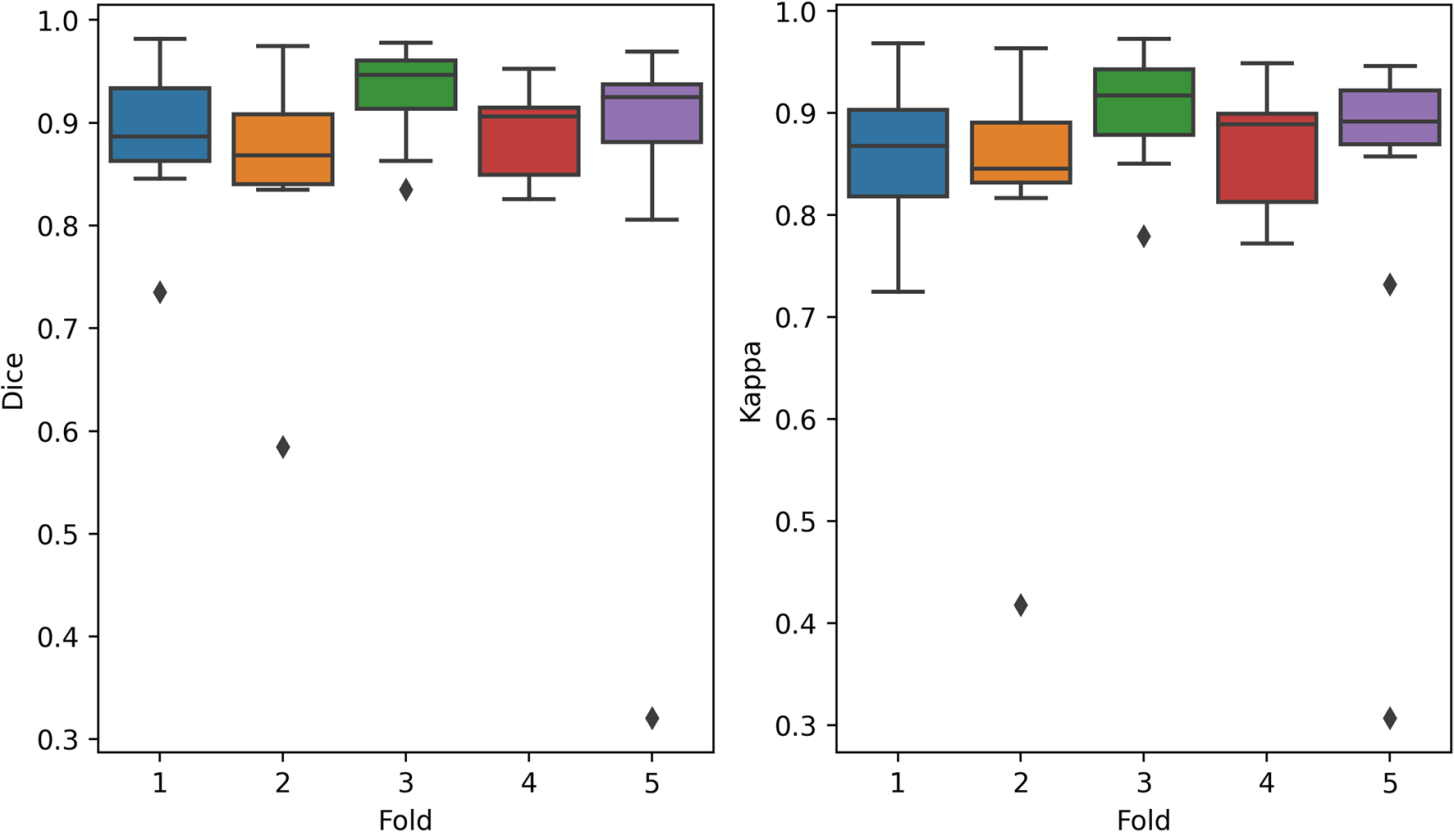
Distribution of the Dice and kappa scores in fivefold cross validation. The line within the box corresponds to median value, the range of each box to Q1-Q3 range (25–75% of scores) and whiskers to lowest and highest observations excluding outliers (individual points).

The proposed method showed stable and high performance in fivefold cross validation with average Dice scores ranging between 0.853 and 0.928. Similarly, Cohen’s Kappa scores ranged from 0.819 to 0.902 (Table 1, Fig. 3).

**Figure 4:**
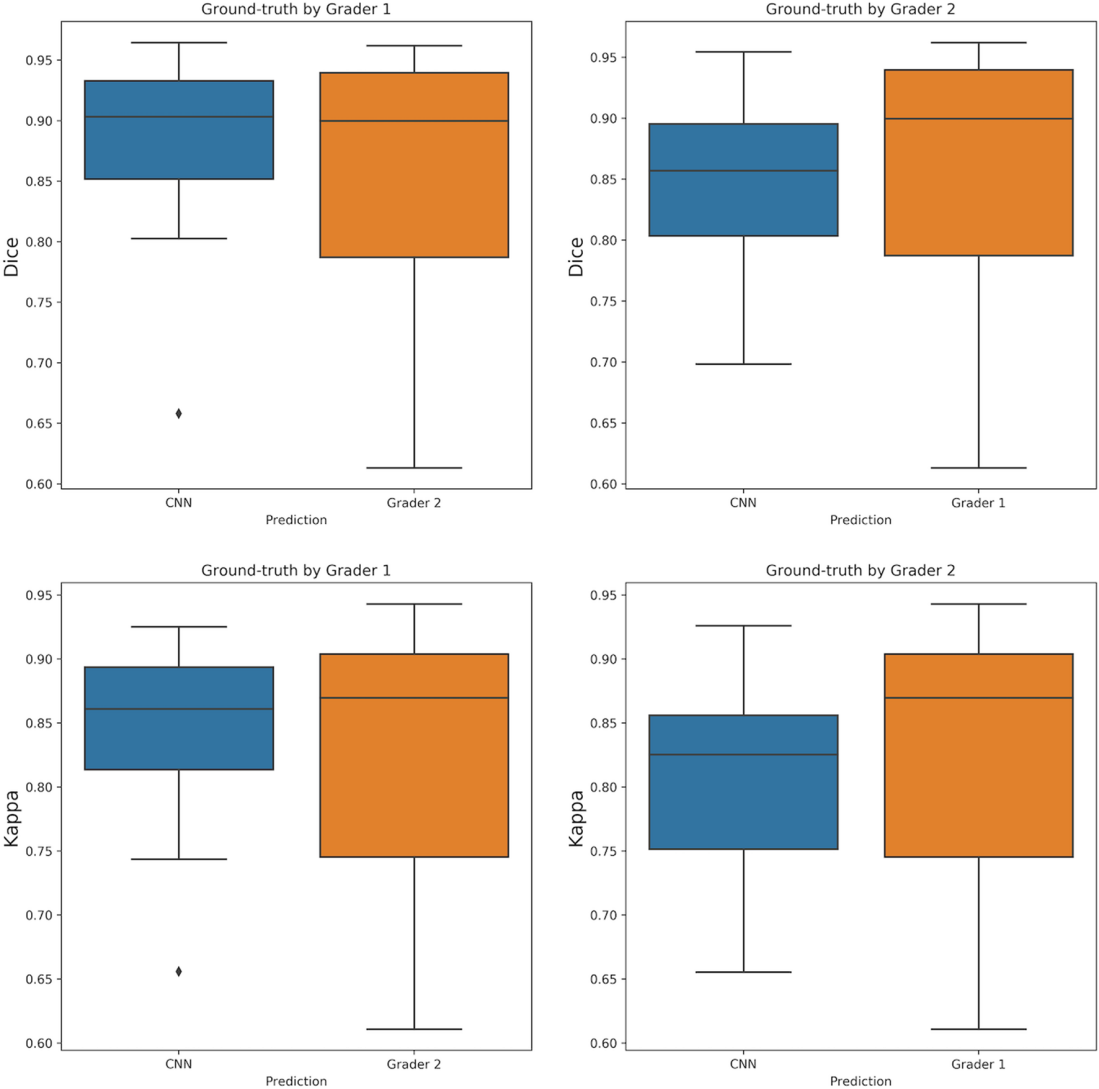
Distribution of the Dice and kappa scores per test volume when using ground-truth provided by either of the graders. The performance of our method matches the level of agreement between the two graders.

**Figure 5:**
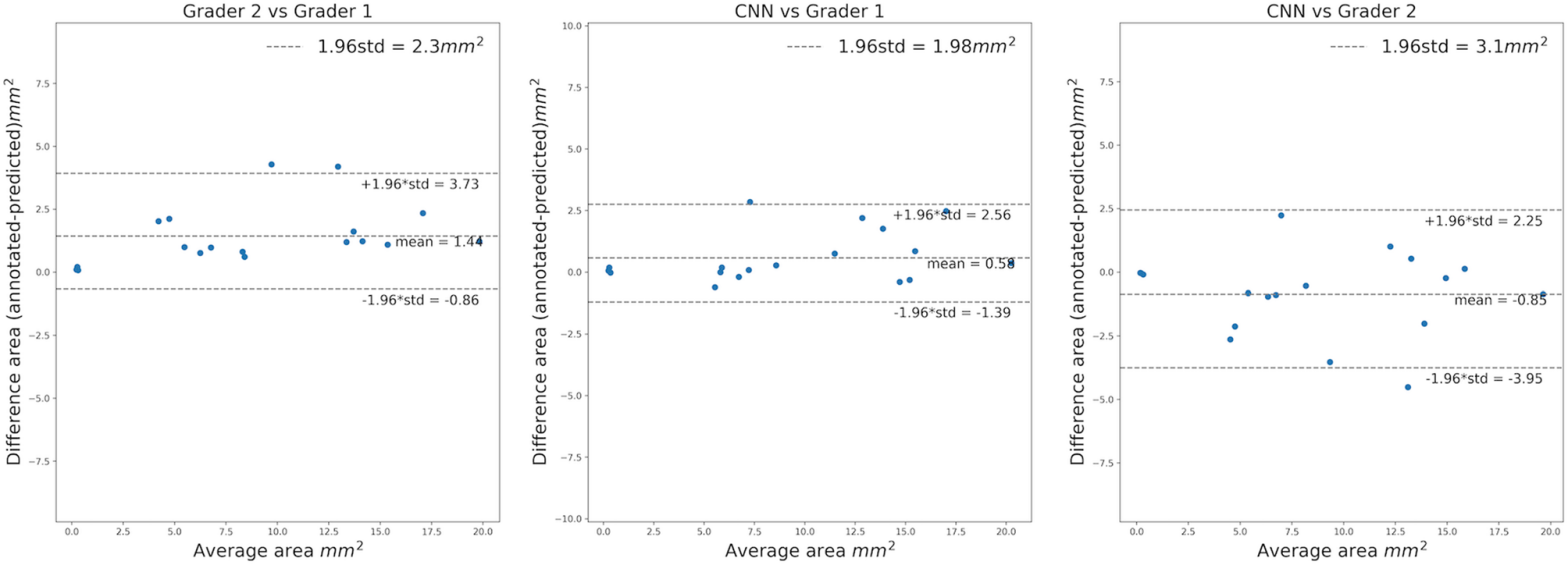
Bland–Altman plot illustrating RORA area differences between graders and our method.

On the additional test set annotated by two graders, our model achieved a similar performance as the experts between themselves. The average Dice score of the CNN was 0.881 and 0.844 compared with Expert 1 and Expert 2 as ground truth, respectively. In comparison, the average Dice score between the experts themselves was 0.863. Most of the test volumes obtained Dice score over 0.85 with respect to Grader 1 and over 0.80 with respect to Grader 2 (Fig. 4). Additionally, the CNN showed high precision (0.928) with respect to Grader 1, and a high recall (0.915) with respect to Grader 2. RORA was not detected in any of the healthy test cases. Figure 5 shows several *en-face* examples of the CNN predictions and manual ground-truth.

The model trained without layer prior achieved lower Dice scores with respect to both graders (0.841 and 0.831) (Table 2). While it improved the overall precision, the recall dropped significantly compared to our proposed approach.

**Figure 6:**
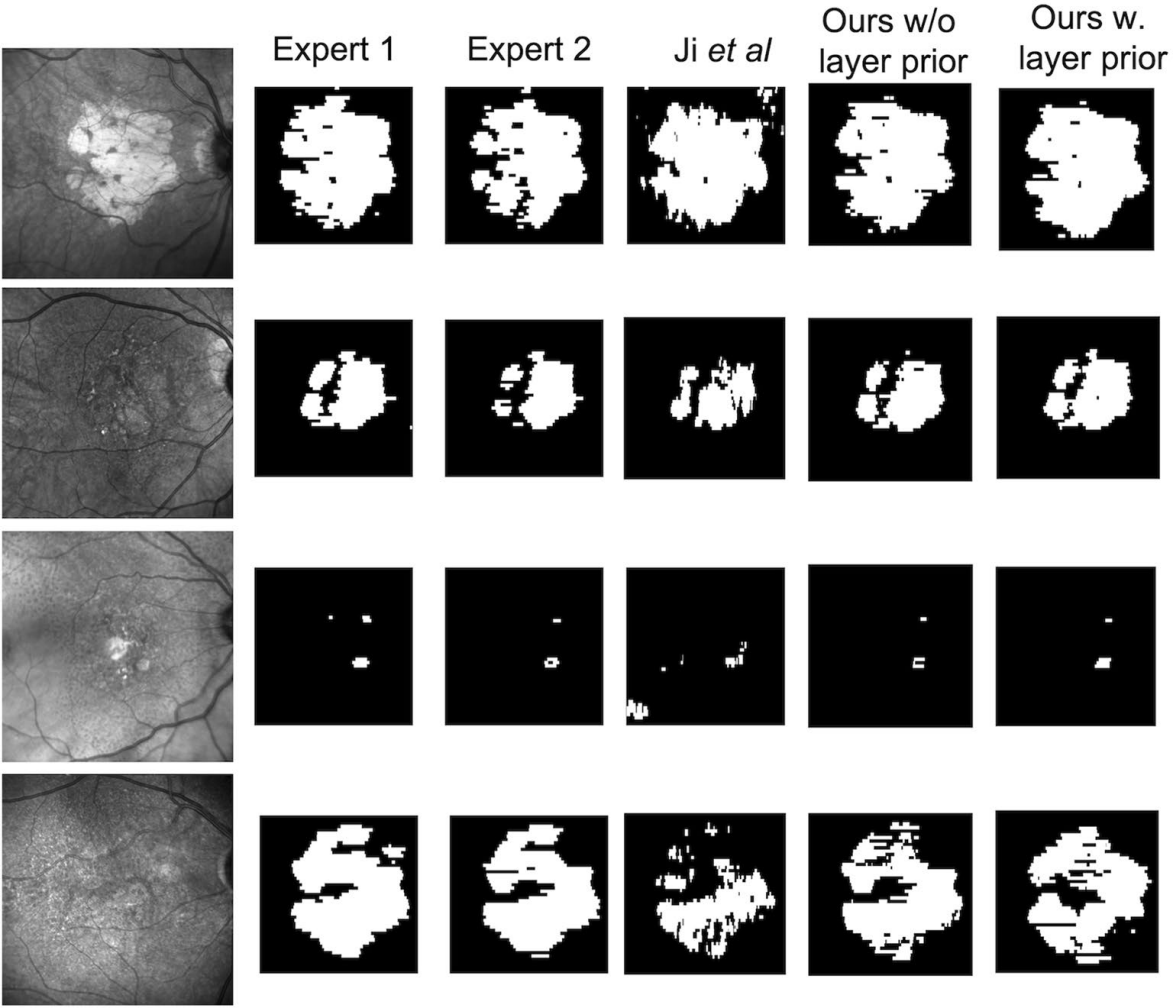
Examples of en face RORA segmentation. From left to right: a corresponding infra-red fundus image, RORA annotated by Expert 1, Expert 2, predicted by the method of Ji et al.^12^ and predicted by our algorithm trained without and with layer prior. Our automatic segmentation approach was able to consistently segment a wide range of atrophic lesions and detected cases where hypertransmission was not clearly visible in infra-red images.

The method of Ji et al.^12^ achieved lower performance than our model, with an average Dice score of 0.716 and 0.677 compared to Grader 1 and 2 respectively. The drop in performance was especially visible in terms of precision. Qualitative examples of predictions obtained with our method (trained with and without layer segmentation) as well as predictions of the method of Ji et al. are shown in Fig. 6.

### Inter-expert Agreement

The level of segmentation agreement between the two experts, as well as that between each expert and the trained models, was computed on the test set in terms of Cohen’s kappa coefficients (Table 2, Fig. 4). The mean Cohen’ kappa coefficient between experts was 0.828. The CNN model showed a mean Cohen’s kappa coefficient of 0.846 and 0.800 with Experts 1 and 2, respectively.

Figure 5 presents Bland–Altman plot for segmented RORA area differences between graders and the automatic method. It indicates that Grader 1 had a tendency to segment a bigger area than Grader 2 and the proposed method closely matched her. Grader 1 was the senior grader (IM), and Grader 2 the junior (YD).

### Complete and incomplete RORA

The prevalence of iRORA lesions in the ground truth of the test set provided by Grader 1 and Grader 2 was 82 and 91 lesions, respectively, based on a b-scan evaluation (horizontal extension of 250 μm). However, on the en face presentation, 78% and 96% of iRORA cases identified by Grader 1 and 2 respectively were in direct connection with a neighbouring cRORA, representing an edge of the RORA lesion rather than an isolated iRORA lesion. Of the remaining iRORA lesions without contact to a cRORA on a neighbouring b-scan (18 cases for Grader 1 and 4 cases for Grader 2), the interobserver agreement was 0.18, and the CNN model was able to identify 16% of iRORA cases delineated by Grader 1 and 25% of iRORA annotated by Grader 2.

## Discussion

The proposed DL method achieved performance comparable to the two clinicians for the detection and delineation of RORA in SD-OCT images among atrophic AMD patients. The disagreement between experts was similar to the difference between CNN and an individual expert. Thus, the CNN performed within the accuracy range that can be found between experts^2^. However, the automatic approach has the advantage of being progressively more consistent with continuous training.

Recent years have seen increased interest in application of automated methods for disease identification and pathology segmentation in OCT imaging. De Fauw et al.^17^. introduced an automatic method for making referral decisions for retinal diseases based on the prior tissue segmentation. Apart from GA, other pathologies of interest segmented in OCT include retinal fluids^18–20^ and Hyperreflective Foci^21^. Schmidt-Erfurth et al.^22^ proposed a method for predicting early AMD progression to the wet type with choroidal neovascularization (CNV) or dry type with GA. For those two tasks it obtained AUC of 0.68 and 0.80 respectively. Venhuizen et al.^14^ evaluated a machine learning method to automatically grade AMD stages from OCT scans. Their method achieved sensitivity of 0.982 and specificity of 0.912, which was comparable to agreement between clinicians. However, the ground-truth clinical grading was based on CFP, and the authors noted that in several cases of nascent GA the algorithm assigned higher severity than the graders. This is possibly because of atrophy already seen in OCT but not yet in CFP, which underlines the importance of OCT imaging in atrophy diagnosis. While previous reports incorporating machine learning for atrophic AMD images^8–15,22–25^ mostly used FAF, according to the classic definition of GA, the recent trend for classifying atrophy in AMD is strongly based on SD-OCT, in order to benefit from its structural information^2,3^. Indeed, SD-OCT enables the detection of pathologic changes of specific retinal layers at various stages of the atrophic process, sometimes even before lesions are clinically visible in fundoscopy.

OCT-based detection algorithms mainly rely on choroidal hypertransmission to identify GA. Hu et al.^8^ proposed a method based on the projection image derived from the region between RPE/Bruch membrane and the choroid, obtaining an average Dice score of 0.87. The method introduced by Niu et al.^26^ processed an OCT projection image with an average overlap ratio of 0.81. Zhang et al.^11^ constructed a projection input using the OCT region underneath the Bruch membrane as an input to a multi-scale CNN, achieving an average Dice score of 0.91. These methods used a set of hand-designed heuristic steps to segment atrophy, which commonly do not generalize as well as data-driven approaches. Conversely, Ji et al.^12^ and Xu et al.^13^ used each OCT a-scan as an input to a neural network instead of a projection image. The initial prediction was subject to refining steps, resulting in an overlap ratio of 0.869^12^ and 0.907^13^. Classification of raw a-scans saves the additional step of preparing a slab image, but at the same time it omits the contextual information of a full b-scan. In contrast to previous reports, which used the classic definition of GA based on FAF and hypertransmission^8–13^, our study used the recently proposed classification system based on SD-OCT, including layer specific criteria for RORA and full b-scan context.

The reported expert grading of the presence or absence of cRORA showed great variability^2^. The gradual degenerative process leads to a spectrum of OCT changes within the process, from normal to incomplete, and finally to complete atrophy, which makes a clear cutoff between the definitions challenging. Despite this difficulty, we found an inter-expert agreement in our study of 0.83, and a Dice score of 0.86 between the experts. This number was calculated on segmentation agreement, which is more challenging than mere presence/absence agreement. Given the high reported variability^2^ we consider the inter-expert agreement found herein to be satisfactory. The CNN segmentation within the range of the inter-expert variability can be considered to be within human-grade annotation performance. Furthermore, the results of cross validation were comparable to the performance on a separate test set and within the agreement between graders, showing stability of the proposed method.

**Figure 7:**
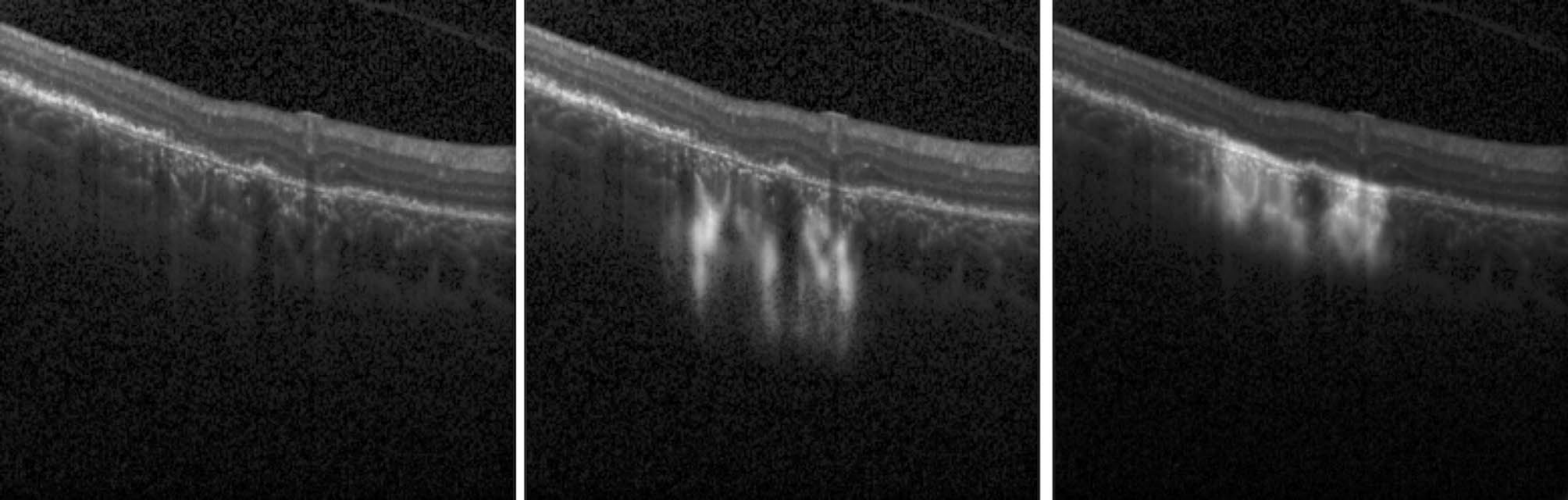
Comparison of outputs obtained with models trained without and with layer information. Left: input b-scan, middle: output of CNN trained *without* layer prior, right: output of CNN trained *with* layer prior overlaid on input b-scan. The network trained using only maximum probability projection focuses mostly on increased hypertransmission in atrophic regions, while the other model also looks at RPE status.

Adding retinal layer information during training increased the algorithm test performance. While the method trained without layer prior still achieved satisfactory results, a significant drop in recall was observed. Figure 7 illustrates the fact that our model looks at the most relevant image regions for RORA definition (RPE and choroid), while the approach trained without layer prior detects primarily increased hypertransmission. While hypertransmission is an important sign of RORA, it is not a sufficient criterion in itself, and therefore the investigation of the RPE region is important.

Applying the method recently proposed by Ji et al.^12^ achieved lower performance. Possible reasons include the facts that their method used individual a-scans as input, did not take into account contextual information on b-scans and that the compared baseline did not incorporate layer information, resulting in lower recall in the atrophic regions where RPE was still relatively intact (Fig. 6, last row).

In general, our method showed higher recall than precision (Table 1). The likely cause is the maximum probability projection, which was applied to obtain en face output and may bias the error towards false positives and over-segmentation. Manual inspection of the outliers seen in Fig. 3 showed that in several cases the model detected RORA in the regions with existing drusen or elongated, flat PED as well as strong hypertransmission in the whole choroid (SI, Fig. S1). Enriching the train set with more OCT scans containing drusen may improve the performance in such cases.

For the differentiation between cRORA and iRORA, several problems became evident. First, the iRORA lesions were infrequent in our cohort, possibly related to the advanced stage of atrophic AMD. Indeed, our inclusion criteria were orientated on the presence of GA which largely corresponds to cRORA. Second, some small b-scan lesions (< 250um) were edges of cRORA lesions rather than isolated, true iRORA lesions. Third, the definition based on size resulted in disagreement of classification due to very small size differences between graders. For all of these reasons, we described the CNN performance with respect to iRORA with caution.

To study the generalizability of our method, we fully separated the OCT patient sets used for the training and testing phases as well as in folds of cross validation, avoiding intra-eye, and intra-patient correlation. Thus, the result of high precision by the algorithm with the test set demonstrates the algorithm’s ability to correctly analyze the criteria for RORA and apply these criteria for cases it had not seen before, including healthy scans.

There are some limitations to our study. Although the number of b-scans included is high, we acknowledge that the number of test eyes was limited. An approach with fewer b-scans per eye and larger number of eyes, would have precluded an *en face* area evaluation of the CNN model predictions. In addition, a wide range of atrophy presentations was included, from small to large lesions, from sharply demarcated to ill-defined RORA borders, from unifocal to multifocal to confluent to diffuse regions (Fig. 6). Thus, we believe that the included spectrum is representative. Moreover, the results of cross validation were in line with the performance on the separate test set, which further shows robustness of the proposed approach.

We acknowledge that the selection criteria of atrophy visible on FAF and SD-OCT may have led to exclusion of earlier stages of atrophy not yet visible on this modality. Therefore, our results cannot be generalized to cases without atrophic signs on FAF. All training and testing scans used in this study were cube scans acquired with the Spectralis OCT machine. While adequate augmentation and resizing techniques should cover at least part of the image appearance variability, inclusion of other manufacturers would potentially increase the proposed method’s robustness and allow us to test its generalizability properties. Similarly, the applicability to neovascular cases has not yet been investigated. Finally, the number of human experts was limited to two. A ground truth with more graders would cover a larger part of the spectrum of inter-expert variability.

Future research should focus on the applicability of such algorithms on a larger spectrum of diseases, including earlier but also neovascular stages. Furthermore, recognition of more stages in the development of atrophy would be valuable.

In conclusion, in this paper we proposed a DL model for the automated detection of atrophy in SD-OCT images. It takes into account the structure of RPE and the choroid, which is in line with the new RORA definition. The results showed excellent performance of the model for RORA identification and measurement on SD-OCT scans. The advantages of this algorithm and future developments include consistency of the algorithm, and rapid calculation of the atrophic area in AMD, all based on the widely available imaging generated with SD-OCT. This might become a useful tool for atrophy measurement and grading, particularly with regards to the difficulty to achieve consensus between human experts. Furthermore, early identification of atrophy could allow patients to be properly staged, enable appropriate monitoring of atrophy, and support new therapeutic development approaches.

## Methods

### Subject and data recruitment

SD-OCT scan volumes were extracted from an existing image database of the Medical Retina Department at Jules-Gonin Eye Hospital. Included were AMD eyes with atrophy on fundus autofluorescence and SD-OCT Spectralis scans (Heidelberg Engineering, Germany; at least 49 b-scans, 6 × 6 mm), and the absence of exudative signs (past and present examinations). Exclusion criteria were a history of retinal treatment, evidence of neovascularization, RPE tear, poor image quality (Heidelberg Spectralis quality level 20 or below, according to the inbuilt quality grading system), or confounding retinal pathologies. A random selection of 57 patients resulted in the recruitment of 62 eyes with fulfilled inclusion and exclusion criteria. The study was approved by the cantonal commission for ethics and human research committee—Vaud (CER-VD 2017-00493) and was performed according to the ethical standards set by the Declaration of Helsinki. Informed consent was waived by the cantonal commission for ethics and human research committee—Vaud (CER-VD).

### Atrophy segmentation and criteria

The entire sample of SD-OCT volumes were separated into three independent sets: a training set (2301 b-sans), a validation set (256 b-scans), which together were obtained from 44 eyes and constituted a development set, and a separate test set from 18 eyes (1038 b-scans). Volumes from the training set were sampled from different patients than volumes from the test set. All training SD-OCT scans were annotated per b-scan indicating the extent of atrophy (definitions below), by two readers (YD and IM). For the test set the experts worked completely independently.

For image segmentation and annotation, the OCT volumes were imported into the OmniViewer software (OmniViewer, RetinAI, V2019.4.0). RORA was identified on each b-scan using bounding boxes to delimit the lateral borders of atrophy, with the RORA zone at its interior. The vertical span of the annotated bounding box was not considered though, as the aim was to evaluate RORA in an *en face* (i.e. projection) view. RORA was defined according to the consensus definition by the Classification of Atrophy Meeting (CAM) Reports as a zone of choroidal hypertransmission and absent or attenuated RPE, with evidence of overlying photoreceptor degeneration^2,3^. In complete RORA (cRORA), the width is larger than 250 μm while in incomplete RORA (iRORA) the width is less than 250 μm. Examples of annotations are shown in Fig. 1A–D.

### Automated segmentation method

The goal of this study was to predict atrophic signs in the retina using 3D SD-OCT volumes. To this end, a CNN was trained, whose input was a single 2D b-scan (a slice of 3D volume on the fast acquisition axis) and the output was a corresponding 2D RORA probability mask (Fig. 2A).

As it is known that RORA affects the RPE and the outer retinal layers, we used retinal layer information as a prior in order to focus the network attention on the photoreceptors and the RPE region during training. Additionally, hypertransmission in the choroid motivated the inclusion of the choroidal region as a region of interest. To this end, every training b-scan was first automatically segmented using the approach of Branch Residual U-shape Network^27^ to obtain an RPE and choroid segmentation mask. Next, it was used to define the vertical extent of the atrophy ground-truth (Fig. 2A). By constructing the training ground-truth where horizontal extent is constrained by RORA width and the vertical dimension by the relevant retinal layers, we force the network to put greater attention to the affected retinal regions, which are emphasized by the latest RORA definition. The retinal layer segmentation was only applied in the training phase to compute the training loss but was not required at inference time.

The encoder part of our U-Net style network was based on the EfficientNet-b3 architecture^28^ pre-trained on ImageNet—a large set of natural images. During training we sampled batches of 32 b-scans and used the Adam optimizer^29^ with a learning rate of 0.000025. We optimized a binary cross entropy loss with focal loss^30^ gamma exponent of 2 for a total number of 50 epochs. We used common data augmentation strategies such as horizontal flip, scaling (in the range 0.8 to 1.2), adding gaussian blur and additive random noise. The model was developed using the Pytorch library^31^. The learning curves for training and validation sets are available in SI, Fig. S2.

### Outcomes and statistical evaluation

The CNN performance was first evaluated with fivefold cross validation using the development data (44 OCT scans of 44 eyes/39 patients). Next, the model was retrained using all available training data and tested on the separate test set (18 OCT scans of 18 patients), where each scan was annotated by two experts. In order to obtain *en face* predictions, the network output was projected using the maximal atrophy probability along the vertical direction, and thresholded at 0.5.

Outcome measures included the inter-expert agreement (Cohen’s kappa coefficients) as well as the sensitivity, precision and the Dice score of the *en face* atrophy segmentation. All metrics were computed per test volume/scan by aggregating b-scan predictions from the given scan.

To investigate the influence of the retinal layer prior in training, the experiment was repeated without including a photoreceptors-RPE-choroid mask. In that case the loss function was computed in 1D by taking the maximum probability projection along the b-scan’s vertical axis (Fig. 2B). Additionally, we compared our method to the one proposed by Ji et al.^12^, which similarly to us and in contrast to other methods did not perform image projection prior to feeding it into the network, but unlike us did not use any layer focusing mechanism during training. Finally, to study the bias of the proposed model and the range of error values, we computed Bland–Altman plots for the RORA area.

The definition of incomplete RORA (iRORA) as a presumed precursor of complete RORA (cRORA) lesions, differs from the cRORA definition by its size smaller than 250 μm. Accordingly, the expert annotations were subclassified as either iRORA or cRORA by measurements on the horizontal b-scans. The prevalence of iRORA lesions and their relationship to cRORA was analysed in a descriptive way. The CNN performance for iRORA was calculated as the discovery rate.

## Supplementary Information

Supplementary Information.

## Data Availability

The datasets generated during and/or analyzed during the current study are not publicly available due to privacy constraints. The data may however be available from the Service of Medical Retina at the University Eye Hospital of Lausanne subject to local and national ethical approvals.

## Code availability

The Machine Learning models described in this work were developed using the Pytorch library. The performance analysis was conducted using code developed in Python.
