# Supplementary material for: Fully-automated atrophy segmentation in dry age-related macular degeneration in optical coherence tomography
Source: Sci Rep. 2021 Nov 8;11:21893. doi: 10.1038/s41598-021-01227-0 (PMC8575929; doi:10.1038/s41598-021-01227-0)
Supplement: Supplementary file 1 — Supplementary Information. [file 41598_2021_1227_MOESM1_ESM.docx]

# Supplementary Information

**
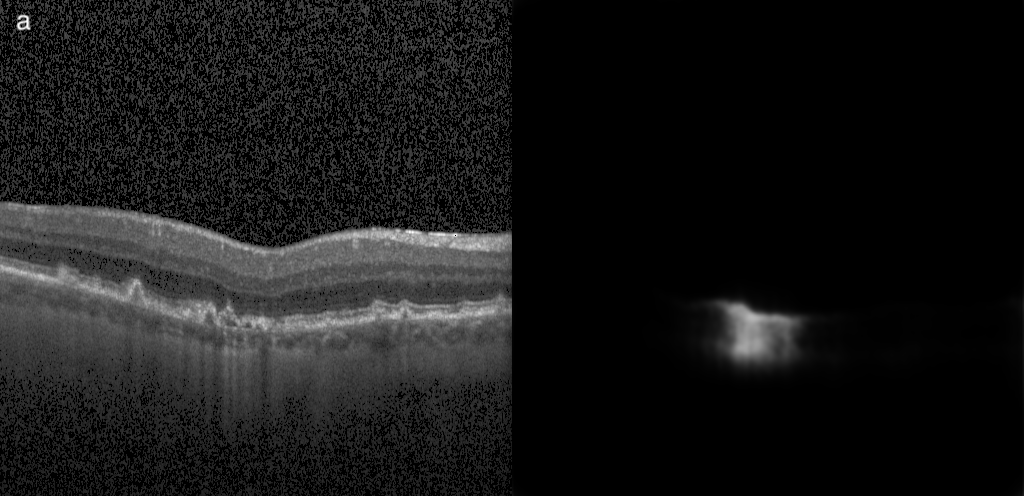
**

**
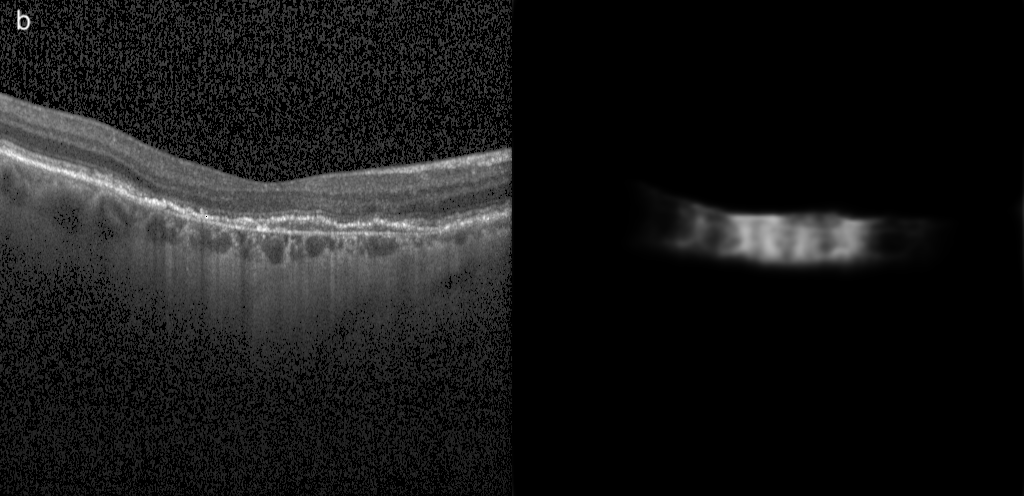
**

**
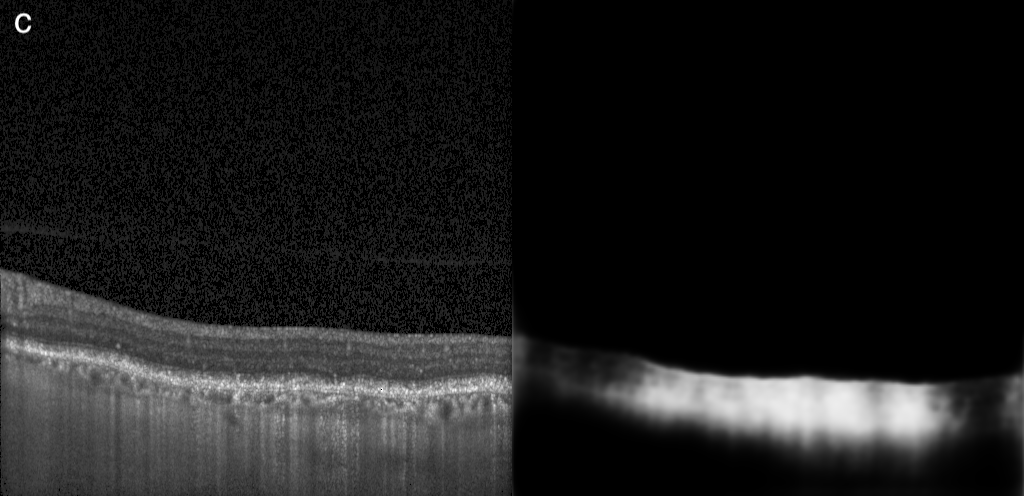
**

**FIGURE 1.** Examples of false positive RORA predictions of our method (a) in the presence of drusen (b) in the presence of flat PED (c) with strong diffuse hypertransmission. In all three cases no RORA was manually annotated.


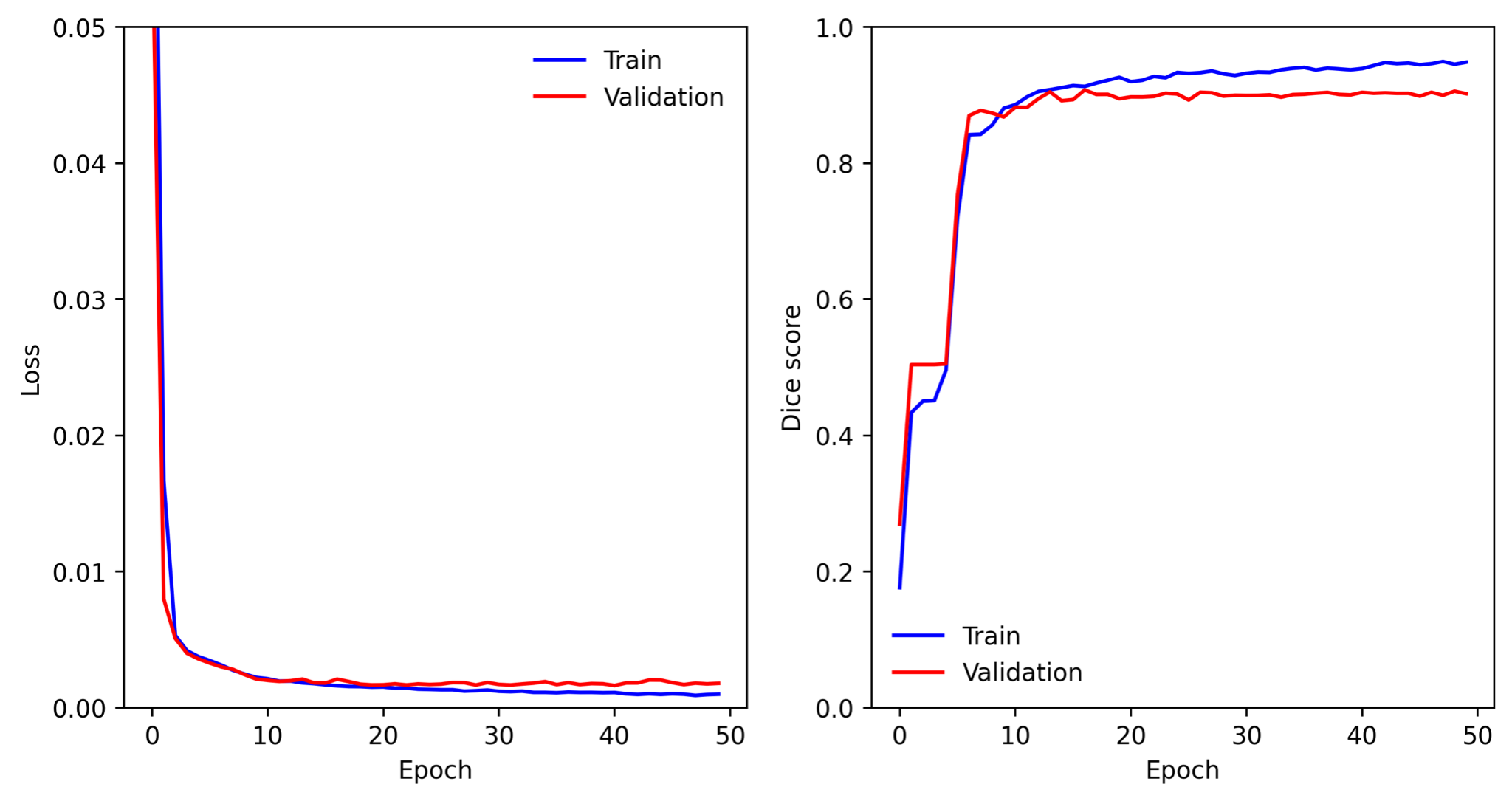


**FIGURE 2.** Learning curves for training and validation sets, based on the loss value (binary cross entropy with focal loss exponent of 2) and Dice scores.
